# Review of Biosensors Based on Plasmonic-Enhanced Processes in the Metallic and Meta-Material-Supported Nanostructures

**DOI:** 10.3390/mi15040502

**Published:** 2024-04-06

**Authors:** Sneha Verma, Akhilesh Kumar Pathak, B. M. Azizur Rahman

**Affiliations:** 1 School of Natural and Environmental Sciences, Newcastle University, Newcastle upon Tyne NE1 7RU, UK; 2 Center for Smart Structures and Materials, Department of Mechanical Engineering, Northwestern University, Evanston, IL 60208, USA; akhilesh.pathak@northwestern.edu; 3 School of Science and Technology, City University of London, London EC1V0HB, UK

**Keywords:** surface plasmon resonance, localized surface plasmon resonance, nano-structured sensors, single/paired nanoparticles, plasmonics, sensitivity, hybrid nanostructures, microfluidic channels, polymer-based sensors, stacked sensors

## Abstract

Surface plasmons, continuous and cumulative electron vibrations confined to metal-dielectric interfaces, play a pivotal role in aggregating optical fields and energies on nanostructures. This confinement exploits the intrinsic subwavelength nature of their spatial profile, significantly enhancing light–matter interactions. Metals, semiconductors, and 2D materials exhibit plasmonic resonances at diverse wavelengths, spanning from ultraviolet (UV) to far infrared, dictated by their unique properties and structures. Surface plasmons offer a platform for various light–matter interaction mechanisms, capitalizing on the orders-of-magnitude enhancement of the electromagnetic field within plasmonic structures. This enhancement has been substantiated through theoretical, computational, and experimental studies. In this comprehensive review, we delve into the plasmon-enhanced processes on metallic and metamaterial-based sensors, considering factors such as geometrical influences, resonating wavelengths, chemical properties, and computational methods. Our exploration extends to practical applications, encompassing localized surface plasmon resonance (*LSPR*)-based planar waveguides, polymer-based biochip sensors, and *LSPR*-based fiber sensors. Ultimately, we aim to provide insights and guidelines for the development of next-generation, high-performance plasmonic technological devices.

## 1. Introduction

**Figure 1:**
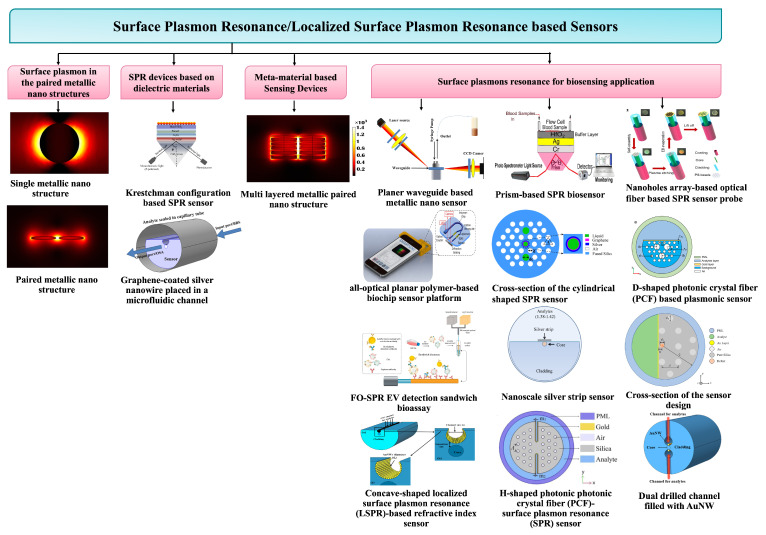
Schematic diagram of the layout of the review paper.

Surface plasmons (*SPs*) have already received huge interest in recent years due to the sub-wavelength spatial distribution of the modal field and their potential to significantly improve light–matter interactions [1,2,3,4,5,6]. Surface plasmons (SPs) [7] represent coherent electron oscillations occurring at interfaces with materials exhibiting positive and negative real parts of dielectric functions. Localized and propagated SP polaritons (*SPPs*) are two main types, with *SP* significantly enhancing photon–material interactions in fields like plasmon-enhanced fluorescence, Raman spectroscopy, and more. The rising popularity of paired gold nanoantennas stems from their versatile applications in optical bio-sensing, particularly leveraging localized surface plasmon resonance (*LSPR*) [8] for biosensing applications [9]. *LSPRs*, known for their high electromagnetic field confinements, find applications in biomedical detection [10], photovoltaic cells [11], spectroscopy [12], energy generation [13], and disease therapy [14]. Interactions with surface plasmons significantly enhance the fluorescence signal, offering a powerful approach to boost excitation rates and increase quantum yield for improved detection in chemical and biological assays. Plasmon-enhanced fluorescence (PEF) on metallic surfaces, also known as metal-enhanced fluorescence (MEF), accelerates detection times and enhances sensitivity in fluorescence-based analytical technologies [15]. Nanotechnology’s efficiency in scattering, absorption, and reflection at the nanoscale has fueled its popularity. In biomedical diagnostics, optical nanomaterial sensors should ideally be affordable, simple to operate, and provide steady, repeatable data. This research benchmarks effective refractive index sensors based on nanostructures. Industrial applications, like photonic device development, involve time-consuming stages, prompting the use of mathematical simulations and software packages. The accuracy of these modeling tools has increased with computing technology. While 2D computations clarify basic concepts, comprehensive 3D simulations are crucial for practical devices. The finite element method (*FEM*) [16] is often employed for studying the plasmonic response of 3D nanostructures. To address computational inefficiencies, an in-house *FEM* model was developed with varying mesh resolutions. Despite supercomputing availability, optimizing plasmonic nanostructured device parameters remains time-consuming due to the *FEM* approach’s evaluation time. Nanostructures, multilayered nanostructures, dual-drilled channels, filled sensor microfluidic sensors, shaped PCR sensors, and metamaterial structures collectively contribute to a revolution in sensing applications. The integration of these advanced technologies offers unprecedented benefits in precision, sensitivity, and speed for various sensing platforms. These innovations enable the detection of minute quantities of substances, enhancing the accuracy of diagnostics and environmental monitoring. Multilayered nanostructures provide a versatile platform for diverse sensing modalities, while dual-drilled channels and filled sensor microfluidic systems facilitate efficient sample handling and analysis. Shaped PCR sensors offer rapid and specific nucleic acid amplification, crucial in molecular diagnostics. Metamaterial structures further extend the sensing capabilities by manipulating electromagnetic waves, opening avenues for novel sensing modalities. In essence, these cutting-edge technologies collectively empower sensing applications with enhanced performance, enabling breakthroughs in healthcare, environmental monitoring, and beyond. The detailed schematic in Figure 1 shows the structure of the sensing application with efficient sensing performance based on structural dimensions.

The review article is organized as follows: In Section 2, we delve into the Principle of Surface Plasmon Resonance, elucidating its occurrence following the interaction with electromagnetic waves. Section 3 focuses on surface plasmon resonance in metallic structures, offering comprehensive insights. Moving forward, Section 4 explores the enhancement of SPR device performance through the integration of dielectric materials. Section 5 is dedicated to metamaterial-based sensing devices, highlighting their improved sensing capabilities. In Section 6, the discussion centers on surface plasmons employed in biosensing applications. Section 7 probes into the future of plasmonic sensors, presenting potential developments and advancements. Finally, Section 8 serves as the conclusion, summarizing the key findings and implications of the presented review article.

## 2. Principle of Surface Plasmon Resonance Occurrence after the Electromagnetic Wave Interaction

**Figure 2:**
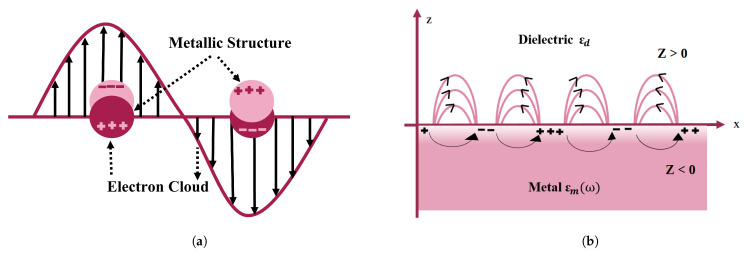
Pictorial depiction of surface plasmon resonance (SPR) in gold nanoparticles. (**a**) Schematic of a propagating surface plasmon polariton and (**b**) localized surface plasmon resonance.

SPR is a viable methodology for gaining insights into the optical characteristics of nanomaterials. SPR-based nanosensors are a configurable innovation for biological modeling approaches. SPR is associated with charge density oscillations at the interface between a metallic layer and a dielectric layer [17] (shown in Figure 2a) because it responds to the optical characteristics of the metal nanostructure [18] and environmental variations. As a consequence, biomoleculars are extremely sensitive to plasmon resonance, obviating the need for exogenous biomolecular labeling [19]. SPR can form in these circumstances [20].

Surface plasmons are classified into two categories on the nanometer scale: localized and propagating plasmons. The aggregate synchronized electromagnetic oscillation is restricted at the subatomic particle surface in the first example, forming an electromagnetic field surrounding it that is referred to as localized surface plasmon resonance (LSPR) [20,21]. In the second scenario, surface charge oscillations interface with electromagnetic waves or incoming photons, propagating along the metal–dielectric interface; these can be defined as surface plasmon polaritons (SPPs) [22]. This is restricted to a two-dimensional environment [23] because SPPs do not couple to EM illumination at the flat metal and vacuum interface. Gratings or prismatic matching techniques are used to create energy coupling [22]. However, SPP propagation is hampered by damping, which becomes a major issue when it is used in sensors, nanocircuits, and plasmonic lasers [24]. LSPR and SPP are depicted schematically in Figure 2. When electromagnetic waves propagate in a transverse direction known as surface plasmon waves (SPWs) [22]. SPWs might be either radiative or nonradiative [25]. With planar electromagnetic waves, coupling occurs, resulting in visual phenomena such as transitional radiation and plasma resonance absorption [24]. The frequency of LSPR varies depending on the material type (platinum, gold, silver, etc.). As a result, minor changes in the surrounding dielectric environment, such as molecular adsorptions on the surface of the nanoparticle, impact the frequencies of LSPR, which may be seen as light scattering and absorption frequency shifts. These shifts can then be readily converted into simple optical transmission or reflection observations with great spectrum precision, allowing nano-sized LSPR systems to be used as excellent sensors for chemical and biological analyte detection.

## 3. Surface Plasmon Resonance in the Metallic Structures

Gustav Mie described the light-matter interaction in metal nanoparticles in 1908 [26], which results in a collective oscillation of free electrons about the nanoparticle structure [27,28,29]. For a spherical metal nanoparticle, when the size is much smaller than the wavelength of the incident electromagnetic field, the plasmon oscillation is dominated by the dipolar mode with a polarizability α given by [28]:

(1) $$\begin{array}{c} \alpha =3\varepsilon_{0}V\frac{\varepsilon -\varepsilon_{m}}{\varepsilon +2\varepsilon_{m}}\;\;\;\;\;\;\;\;\; \end{array}$$

where *V* is the volume of the nanostructure, $\varepsilon_{0}$ is the permittivity of the vacuum, $\varepsilon_{r}$ = $\varepsilon_{r}\left(\omega \right)+j\varepsilon_{j}\left(\omega \right)$ is the dielectric function (in the complex form) of the metal, and $\varepsilon_{m}$ is the dielectric constant of the surrounding medium. The strong plasmonic properties occur due to electromagnetic frequency ω, where $\varepsilon_{r}$ = −2$\varepsilon_{m}$; from this, the surface plasmon resonance (SPR) frequency can be calculated. For silver (Ag), gold (Au), and copper (Cu), the resonance condition is fulfilled in the visible range [28] so it is a promising metal for many optical applications. In addition to significantly increasing the local electromagnetic field at the nanoparticle’s surface, as well as the nanoparticle’s ability to absorb and scatter light at SPR frequencies, the substantial optical polarization correlated with the SPR causes these effects [28,30]. The SPR properties depend on the type and size/shape of the nanoparticle [27,30,31], the enhanced dielectric properties of the surrounding medium [32,33], and inter-nanoparticle coupling interactions [34,35,36], which impart a unique tunability for a variety of optical applications. Sir Paul Drude, in 1900, discussed the behavior of free electrons in metal. This model can be used to find the optical and structural properties of any metallic structure. This approach concentrates on the free electrons that reside in metal and produce surface plasmons; therefore, we could simply compute the surface plasmon resonance using transmission, reflection, and absorption spectra with this framework.

The permittivity function calculated by Drude is given below:

(2) $$\begin{array}{cc} \epsilon_{r} & =\epsilon_{\infty }-\frac{\omega_{p}^{2}}{\omega (\omega +j\gamma )}\;\;\;\;\;\;\;\;\; \end{array}$$

where $\epsilon_{r}$ is relative permittivity, $\epsilon_{\infty }$ is the permittivity of the metallic nanostructure, $\omega_{p}$ is the resonating frequency, known as the real frequency of undamped vibrations of the free atoms. It is defined by $\sqrt{\frac{4\pi Ne^{2}}{m_{0}}}$, where N is conduction electron density and $m_{0}$ is the effective optical mass. γ (free electron oscillations) occur due to damping through electron collisions with collision frequency, shown as $\gamma =\frac{1}{\tau }$, where τ is the relaxation time, which is nearly $10^{-14}$ s. The dielectric functionality may be defined as a mixture of the phase difference between both the driving frequency and the real frequency of the electrons and power losses via dampening when the metal is approximated as a driven, damped oscillator. Taking these considerations into account, the dielectric function may be expressed as a complex having real and imaginary components, as shown in Equations (3) and (4).

(3) $$\begin{array}{c} \varepsilon_{real}=1-\frac{\omega_{p}^{2}\tau^{2}}{1+\omega^{2}\tau^{2}} \end{array}$$

(4) $$\begin{array}{c} \varepsilon_{imag}=\frac{\omega_{p}^{2}\tau }{\omega (1+\omega^{2}\tau^{2})} \end{array}$$

For metallic structures at near-infrared frequencies, when ω>>1/τ, Equations (3) and (4) simplify to [37].

(5) $$\begin{array}{c} \varepsilon \left(\omega \right)=1-\frac{\omega_{p}^{2}}{\omega^{2}}+j\frac{\omega_{p}^{2}}{\omega^{3}\tau }=\varepsilon_{real}^{f}+j\varepsilon_{imag}^{f} \end{array}$$

The actual component is indeed the phasing lag, which would be caused by the retardation of an incoming electromagnetic wave through all the metal and is determined by the metal’s permittivity to photons. Metals have a negative dielectric coefficient at angular frequencies smaller than resonance frequencies. The imaginary component is caused by wave attenuation inside the metal as a result of resistance losses and incident beam absorption. As illustrated in the equation, the complex dielectric function may be expressed in terms of its real and imaginary equivalents to Equation (5).

**Figure 3:**
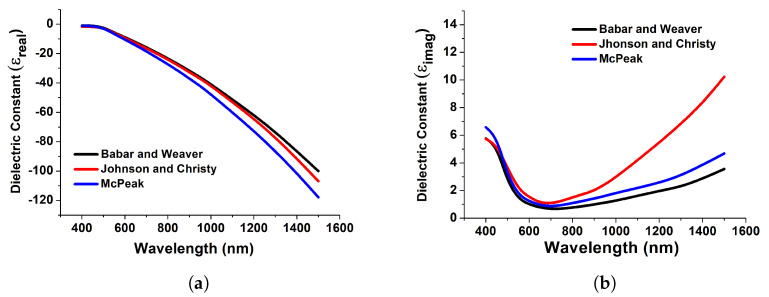
Real (**a**) and imaginary (**b**) counterparts of the dielectric constant of gold obtained from [37].

Figure 3 depicts the computed real and imaginary components of the dielectric permittivity for gold, which have been used to compute the reflection and transmission coefficients. Figure 3 demonstrates the relationship between the actual dielectric permittivity and the wavelengths. Its value decreases across the UV region until it becomes negative in the visible range and remains mostly negative in the infrared spectrum. This electromagnetic feature of the actual dielectric permittivity is responsible for the diverse optical activity of metals, such as the presence of surface plasmon resonance. Resonances are charged particle vibrations inside the atmosphere. Here, in Figure 3, datasets have been collected from Babar and Weaver [37], Johnson and Christy [37], and McPeak [37] for achieving accurate and strong resonance. Furthermore, we have implemented the model in COMSOL Multiphysics by importing these datasets.

An external magnetic field generates polarity proportional to intensity, resulting in polarizability. Equation (1) demonstrates how the form, composition, and environment of the nanoparticles affect the charge density reaction to extrinsic disturbances. Because the polarizability and dielectric coefficient of the metal are frequency-dependent, the polarizability exhibits a resonant amplification as the denominator gets closer to a limit. The Poynting vector of the electromagnetic fields from the nanosphere, which is thought of as an oscillating dipole, may be used to calculate the cross-section [38]. The scattering and absorption cross-sections are given in Equations (6) and (7), respectively.

(6) $$\begin{array}{c} C_{sca}=\frac{k_{0}^{4}}{6\pi }{\left|\alpha \right|}^{2}=\frac{8\pi }{3}k_{0}^{4}r^{6}{\left[\frac{\varepsilon_{m}-\varepsilon_{d}}{\varepsilon_{m}+2\varepsilon_{d}}\right]}^{2} \end{array}$$

(7) $$\begin{array}{c} C_{abs}=k_{0}Im\left|\alpha \right|=4\pi k_{0}r^{3}Im\left[\frac{\varepsilon_{m}-\varepsilon_{d}}{\varepsilon_{m}+2\varepsilon_{d}}\right] \end{array}$$

where $k_{0}$ is the wave-vector of the angular frequency and $C_{ext}$ (the sum of these two cross-sections) provides the resonantly enhanced extinction cross-section, as shown by Equation (8).

(8) $$\begin{array}{c} C_{ext}=C_{sca}+C_{abs}=9\frac{\omega }{c}\varepsilon_{d}^{\frac{3}{2}}V(\frac{\varepsilon_{i}}{{\left(\varepsilon_{real}+2\varepsilon_{d}\right)}^{2}+\varepsilon_{imag}^{2})} \end{array}$$

where ***V*** is the volume of the nanostructure and $\varepsilon_{real}$ and $\varepsilon_{imag}$ are the real and imaginary dielectric coefficients of the metal (Equation (5)). *LSP* mode can be calculated using Equation (5), assuming the metal follows the Drude model $\omega_{LSP}=\frac{\omega_{p}}{\sqrt{2\varepsilon_{d}+1}}$.

### 3.1. Occurrence Surface Plasmon Resonance in the Metallic Structure

#### 3.1.1. Surface Plasmon Resonance in the Single Metallic Nanostructures

**Figure 4:**
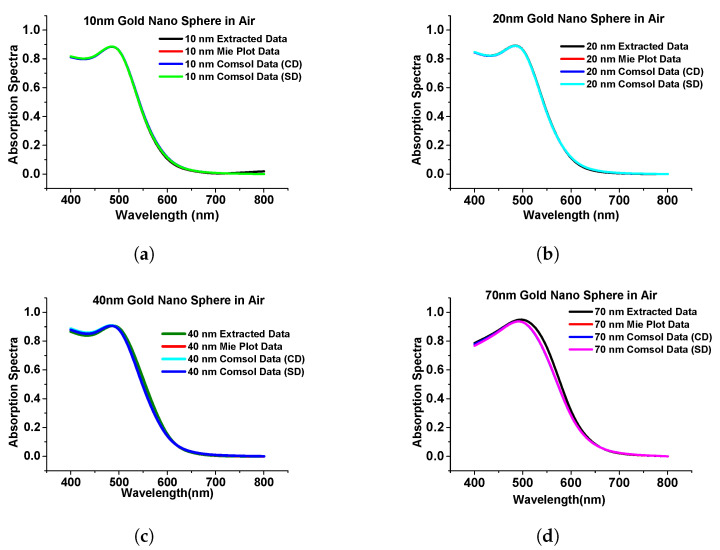
Comparison of simulated results with published work. (**a**) The absorption spectra of gold nanosphere of 10 nm radius. (**b**) Absorption spectra of gold nanosphere of 20 nm radius. (**c**) Absorption spectra of gold nanosphere of 40 nm radius. (**d**) Absorption spectra of gold nanosphere of 70 nm radius. Original results from [39].

In this section, we have optimized the performance of a stand-alone gold nanosphere and benchmarked it with that from Mie theory [39] and calculated the absorption, extinction, and scattering efficiency at a different radius of the sphere when the surrounding medium was air just to observe the performance of the nanostructures. Figure 4 shows the simulated absorption spectra using COMSOL multiphysics of the gold nanosphere (in the Cartesian and spherical domain shown by blue and green curves, respectively) and compared with results from Mie plot theory [40] (also simulated in this work) and the published work [39] is shown by the black curve.

**Figure 5:**
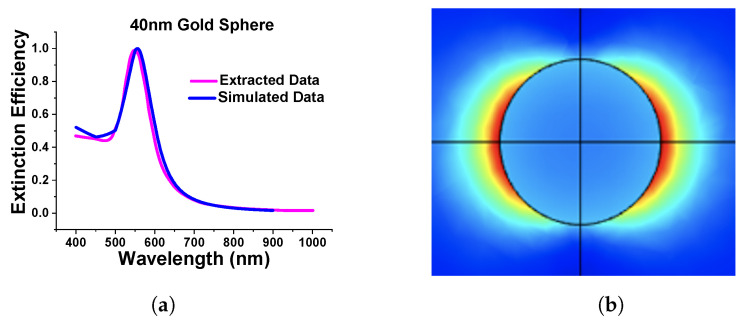
Comparison of simulated and extracted from [41]. (**a**) Extinction efficiency of stand-alone gold nanosphere of 40 nm diameter in water. (**b**) Simulated normalized electric field of nanosphere.

As the above results show excellent agreement on the absorption spectra with different methods, particularly for smaller spheres, it can be said that the designed model works as expected in both Cartesian and spherical coordinate systems as achieved almost similarly concerning the published work. Additionally, we extended the work to a submerged gold nanosphere in water (original design adopted from the [41]). In this work, we have designed a 40 nm radius gold nanosphere that was submerged in the water environment. Here, Figure 5a shows the comparison of our simulated extinction efficiency of the sphere (shown by a blue curve) with the published work (shown by a pink curve).

Figure 5b shows the normalized electric field profiles of the simulated works. For this review paper, we have carried out many simulations to gain more familiarity with the FEM and analyze the accuracy of the generated results.

**Figure 6:**
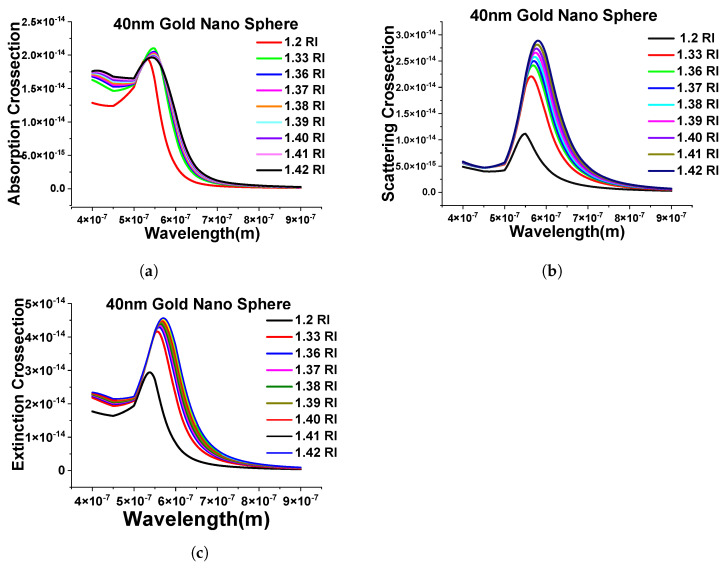
Response of the 40 nm nanosphere in the different surrounding medium. (**a**) Absorption cross-section of stand-alone 40 nm gold nanosphere in mentioned refractive indexes. (**b**) Scattering cross-sections of the same structure. (**c**) Extinction efficiency response of the different refractive indexes (RIs).

Next, we have extended the above-shown results with the evaluation of the performance of different surrounding mediums (from RI 1.2 to 1.42) and simulated the designed FEM model to calculate the absorption cross-section shown in Figure 6a. Here, it can be seen that as the refractive index of the surrounding media is changing, the spectra are shifting toward the higher wavelength, and, similar patterns can be seen while calculating the scattering and extinction cross-section, as shown in Figure 6b,c.

**Figure 7:**
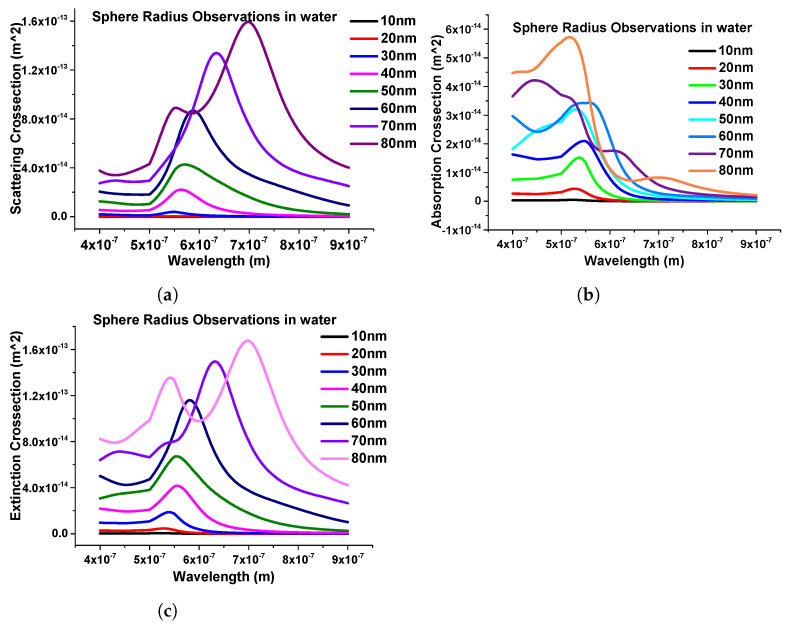
Response of nanospheres submerged in water. (**a**) Scattering coefficients of nanospheres. (**b**) Absorption coefficients of the same structure. (**c**) Extinction coefficient response of nanospheres with different radii.

Furthermore, the effect of the structural geometries of the nanosphere was calculated. To study the structural effect of the nanosphere, the dimension of the nanostructure was changed from 10 nm to 80 nm. In this case, we started from 10 nm radius to 80 nm with a 10 nm step size and calculated the scattering, absorption, and extinction efficiencies, which are shown in Figure 7a–c, respectively.

One important thing here is to observe that as the dimensions of the nanostructure change, the modes start to appear after the 70 nm radius, as shown in Figure 7. It can also be seen that as the radius of the sphere increases, the intensity of the peak increases; hence, from here, it can be observed that the geometrical size plays an important role in obtaining the *LSPRs*.

#### 3.1.2. Surface Plasmon Resonance in Periodic Metallic Nanostructures

After successfully discussing the importance of the single metallic nanostructure, next, we considered the periodic nanodisk placed on a quartz substrate and compared our work with that of Rizzato et al. [42]. The motivation behind this work was to obtain an idea about both the experiment and theoretical calculations. In their work, Rizzato et al. [42] mentioned the surrounding media; however, they used sodium chloride *(NaCl)* in their experiment for the surrounding media. Hence, we simulated the refractive indexes (*RI* from 1 to 1.7 with 0.1 step size) to identify which one is closer to the published result for sodium chloride *(NaCl)* to study the performance of the developed model concerning the published work.

**Figure 8:**
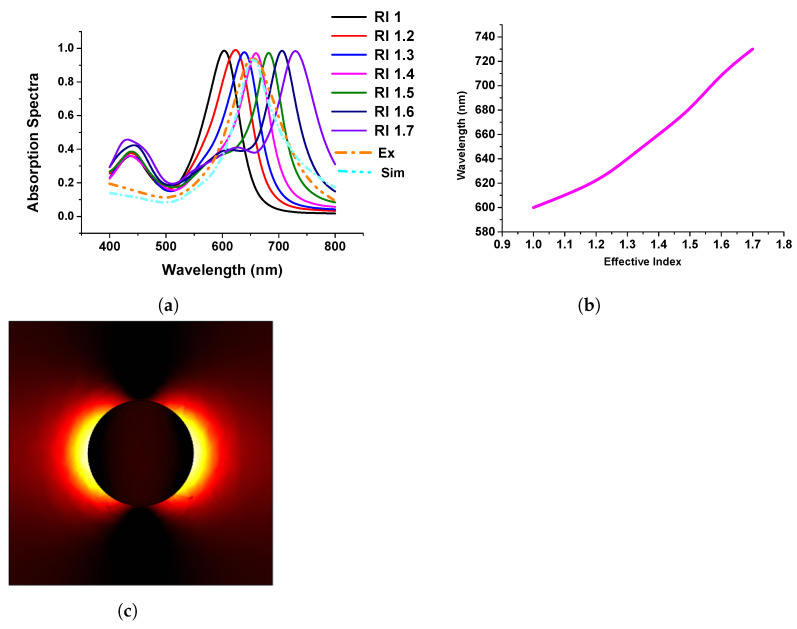
Response of gold nanodisk in different refractive indexes (RIs). (**a**) Absorption efficiency of stand-alone gold nanodisk in different RIs. (**b**) Linear relation with RI and wavelengths. (**c**) Mode profile of normalized electric field.

Figure 8a shows the absorption spectra of the gold nanodisk of 80 nm diameter and surrounding refractive index varied from 1.0 to 1.7. The performance of the developed model was compared with the published experimental and simulation spectra shown by dashed orange and sky blue curves, respectively. From this, it can be stated that when the refractive index of the surrounding media was close to 1.4; in that case, our simulated results agree well with the published work. From these findings, we have benchmarked our modelling works and extended them to visualize the plasmonic wavelength shift, which is plotted concerning the refractive index variation of the surrounding medium, as shown in Figure 8b. From this figure, it can be concluded that the wavelength shift follows a linear pattern when the refractive index changes in the surrounding media. Additionally, Figure 8c shows the electric field distribution of the gold nanodisk along the x−y plane.

#### 3.1.3. Surface Plasmon in the Paired Metallic Nanostructures

**Figure 9:**
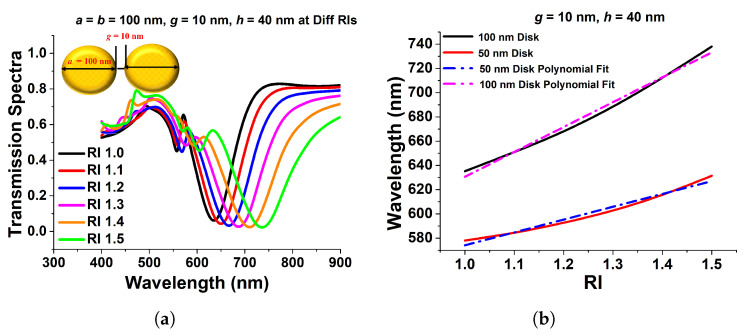
(**a**) Transmission spectra of paired 100 nm circular nanodisk with 10 nm separation distance and 40 nm height. (**b**) Sensitivity and R-square error calculation of 100 nm and 50 nm paired circular disk [43].

Since it is usually recognized that paired nanostructures could increase the field intensity across their gaps, in this section, we will discuss the effect of the paired circular and elliptical disks on the surface plasmon resonance. As a result, the transmission spectra of a 100 nm gold nanodisk surrounded by a material with varied refractive indices are displayed in Figure 9a. One can see that when the refractive index increases, the resonating wavelength shifts to a higher wavelength. The separation gap, *g*, and the height of the disks, *h*, are set to 40 nm and 10 nm, respectively. The resonating wavelength increases when the surrounding refractive index, RI, is increased, as shown in Figure 9b. The slopes of these two curves are 105.79 and 205.18, for diameters 50 nm and 100 nm, respectively, which are used to compute the sensitivities of the coupled circular nanodisks. A nearly linear relationship between wavelength fluctuations and RI variations has also been demonstrated. For 50 nm and 100 nm diameters, the R-square errors were estimated as 0.98915 and 0.9137, respectively, indicating a nearly linear correlation. The acquired sensitivity of the 100 nm coupled disk is bigger than that of the 50 nm paired disk, as illustrated in Figure 9b.

**Figure 10:**
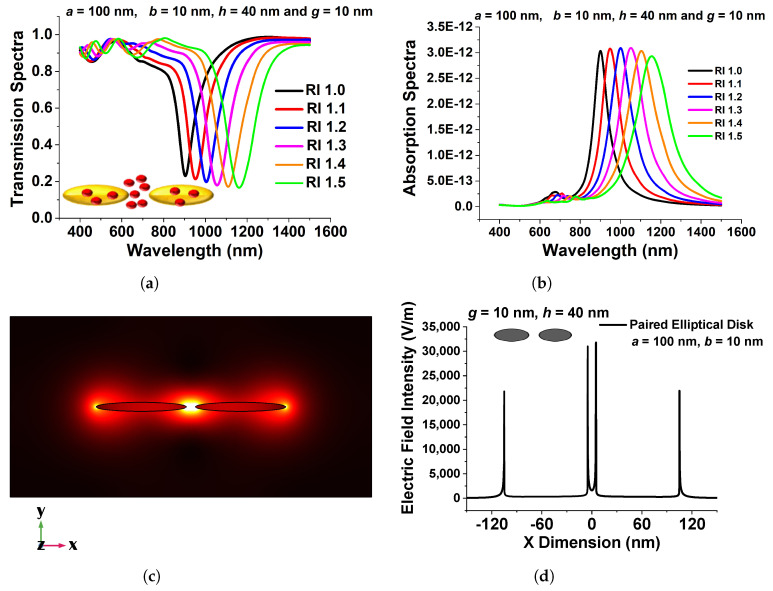
(**a**) Transmission spectra of the optimized paired elliptical antenna with 100 nm, 10 nm major and minor axes, respectively, with 10 nm separation distance *g* and 40 nm height, *h*. (**b**) Absorption Spectra of the same structure. (**c**) $E_{x}$, mode field profile of the optimized paired structure. (**d**) Variation in the electric field along the *x*-direction of the optimized elliptical disk of 100 nm paired circular nanodisk with 10 nm separation distance, *g*, and 40 nm height, *h* [43].

Figure 10a shows transmission spectra for a selected design with various surrounding medium (n). Here, 100 nm, 10 nm, 10 nm, and 40 nm are used as the major axis, *a*, minor axis, *b*, separation distance, *g*, and height, *h*, respectively. Because this design concept exhibits a greater efficient change in resonating wavelength, it could be employed as a refractive index sensor and is a good contender for RI-detecting applications. The narrow band coupled nanostructures’ spectral absorption may also be modified to fit the distinctive absorption spectra of selected RI to identify targeted media in the IR range.

Figure 10b illustrates the absorption spectra of the developed coupled nanoantenna array for six RI values, which validates the observed transmission spectra and demonstrates sensing responses. The resulting electric field intensity in the separation region and extremely high electric field containment in the centre of the coupled antenna is shown in Figure 10c. The intensity of the localized electric field is critical for label-free identification. Because the highest electric field is developed at the centre of the separation gap in the designed structure, such an ultra-strong electric field can be used for sensing applications. Figure 10d depicts the variation of the electric field $E_{x}$ in the *x* direction across the centre of the elliptical antenna pair. Although electron conduction provides an efficient force at the surface of the paired device, the electric field in the separation gap region is significantly enhanced, as shown in Figure 10d. The maximum normalized electric field, shown by a black curve in Figure 10d, reaches up to 35,000 V/m at the inward edge of the paired elliptical disk for *a* = 100 nm, *b* = 10 nm, *g* = 10 nm, and *h* = 40 nm, indicating that the highest field intensity is more than 50% greater in the gap, especially in comparison to the field at the outer edges. It can be regarded as a superior choice for biosensor applications owing to its increased strength of electric field containment. The applied electric field profile shows that by reducing the minor axis, a considerable field increase may be observed at the sharp corner of a coupled structure. This coupling causes the LSPR improvement because the elliptical structures interact more strongly as they approach closer to one another. When the separation distance was larger, the change in transmission and absorption spectra of the resonating wavelength was smaller; therefore, a smaller separation distance is recommended to generate an intense electric field concentration. The maximum localized field intensity forms due to the coupling of surface waves on nanostructures, as shown in the aforementioned figures, and could be an ideal contender for RI sensing applications.

**Figure 11:**
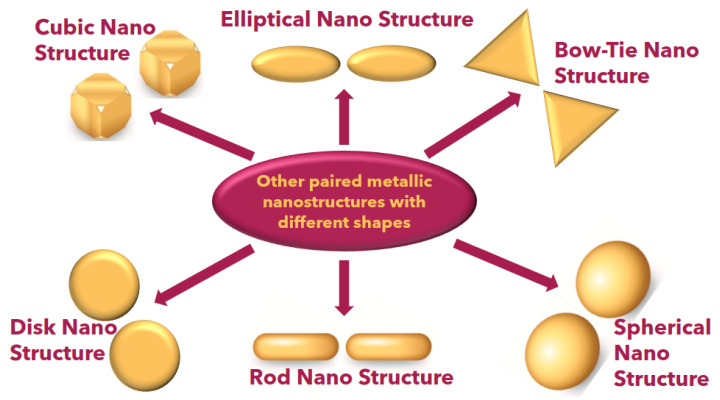
Schematic other paired metallic nanoparticles.

In Figure 11, a comprehensive depiction of diverse paired gold nanostructures is presented, showcasing their unique characteristics based on localized surface plasmon resonance (LSPR). The intricate interplay between the localized electromagnetic fields and the morphology of these paired gold nanostructures is evident in Figure 11, offering a visual representation of the fascinating diversity within this class of nanomaterials. Researchers in the field can glean insights into the nuanced optical responses of these nanostructures, facilitating a deeper understanding of their potential applications and paving the way for advancements in fields such as biomedical sensing, catalysis, and optoelectronics. Table 1 shows the various nanoparticles and their characteristics in various applications.

This table serves as a valuable resource for elucidating the nuanced relationships between different structural shapes and optical behaviour in paired gold nanostructures. Furthermore, in 2003, Beversluis et al. designed surface plasmon-enhanced transitions of gold nanospheres for the visible and infrared photoluminescence continuum [56], and parallelly, Bouhelier et al. simulated elliptical clusters of gold particles for optical devices [57]. Nanospheres have also been used for secure and controllable drug delivery systems, hyperthermia, static/dynamic thrombolysis assessment [58], and also used for drug delivery and therapeutic approaches, which are particularly effective as targeting agents in tumor-bearing subjects [59]. They have also shown promising results for psoriasis treatment, methotrexate drug delivery, and topical therapy in psoriasis patients [60]. In 2017, these nanospheres have also been used to treat cervical cancer, which can help to decrease the death rate in women [61]. In the following years, Ochmann et al. treated single molecule-based point-of-care diagnosis for Zika virus detection [62]. In 2016, Lotscha et al. designed gold nanorods for various biological applications, including cytotoxicity detection [63], and also the colorimetric determination of hypochlorite in water [64], fluorescence enhancement [65], photothermal ablation of tumor cells [66], strain sensing [67], and solar cell applications [68]. Gold nanodisks have been employed for hydrogen sensing [69], detection of PSA cancer markers [70], energy harvesting, spintronics/magnonics, biosensors [42], optical switching [71], medical diagnostics, drug delivery, and chemical sensing [72], as well as other sensing applications [73]. Cesaria et al. designed nanoholes for nano-optical transducer sensing and the integrated/multiple-detection of lab-on-a-chip devices using unconventional lithography [74]. Bow-Tie antenna has shown highly encouraging results in terms of biological sensing and nano-optics applications [75], bioinspired surfaces and dielectric metamaterials [76], and polarimetric optical biosensing [77]. A gold nanostar designed by researchers has been utilized for various applications, such as HeLa cell transfection with PGFP under optimized optoporation conditions [78], singlet oxygen production [79], early cancer detection [80], tumor detection and killing [81], and photothermal therapy, targeted drug delivery, and anti-tumor/anti-bacterial devices [82]. The nanocubic structure also shows a significant role in applications like phenolic biosensors [83], autoantibody detection from body fluid samples [84], cell imaging of human liver cancer cells (QGY) and human embryo kidney cells [85], biology and medicine [86], nanoscale galvanic replacement reactions [87], anticancer natural product [88], plasmonic refractive index sensing [89], photoacoustic imaging-guided radio/photodynamic/photothermal synergistic therapy [90], and photoacoustic imaging of tumor protease capturing the vibrational fingerprints of lipid molecules [91,92]. Various antenna geometries, including gold nanoplates, have been studied for monitoring pH in saliva [93]. Yang et al. have designed gold nanorings for photodynamic cancer therapy [94]. The asymmetric-split ring resonator has been employed for AFM imaging and plasmonic detection, demonstrating an order of magnitude increased sensitivity over non-resonant structures and water treatments [95,96]. A butterfly nanoantenna has been designed for orbital angular momentum (OAM) applications [97]. A diamond-shaped antenna has been fabricated for biotechnology [98], and a mushroom-shaped antenna has shown promising results for refractive index sensing [99]. Dumbbell and parabolic-shaped structures have demonstrated impressive results for photovoltaics, electroluminescence, non-linear optics, and plasmon excitations [100,101]. Zhu et al. [102] observed that as the gap between nanoparticles decreases to the subnanometer scale, quantum mechanical effects, specifically electron tunneling and nonlocal screening, become more pronounced. This implies that at such small distances, the behavior of electrons and their interactions deviate from classical expectations, introducing a new layer of complexity to the understanding of nanogap plasmons. To delve deeper into these phenomena, researchers have undertaken both theoretical and experimental studies to explore and comprehend the implications of these quantum effects in the context of nanoscale plasmonics [102]. Mamiyev et al. [103] have also shown that the oxidation of Au atomic wires on stepped Si(553) surfaces induces minimal impact on plasmonic dispersion, as observed through infrared absorption and electron energy loss spectroscopies. The increase in plasmon energy near k → O is attributed to standing wave formation on small Au wire sections due to introduced O atoms, rather than electronic gap opening, aligning with findings from infrared spectroscopy and electron diffraction. Later on, in 2019, the same group investigated the dispersion in plasmonic resonances refers to the variation in the frequency response of a material’s electronic excitations. In the case of TAPP-Br adsorption, it induces a push-back effect, causing a shift to higher frequencies and enhanced electronic damping, influencing the confinement of free charge carriers in one-dimensional channels and altering the plasmonic signal on gold-doped Si(553)-Au surfaces [104]. Silica-coated noble metal nanoparticles exhibit strong surface-enhanced fluorescence and Raman scattering. To enhance the optical signal effectively, this study employs [Au-Ag alloy NP cluster]$SiO_{2}$ core-shell nanostructures, achieving simultaneous enhancement of Raman scattering and fluorescence emission. Zheng et al. [105] investigates the in situ enhancement comparison of fluorescence emissions and Raman scattering in different types of metal NP agglomeration. The above literature review shows the promising results of strong resonance and field confinement through numerical modeling and experimental investigations in the last few years.

## 4. SPR Devices Based on Dielectric Materials

**Figure 12:**
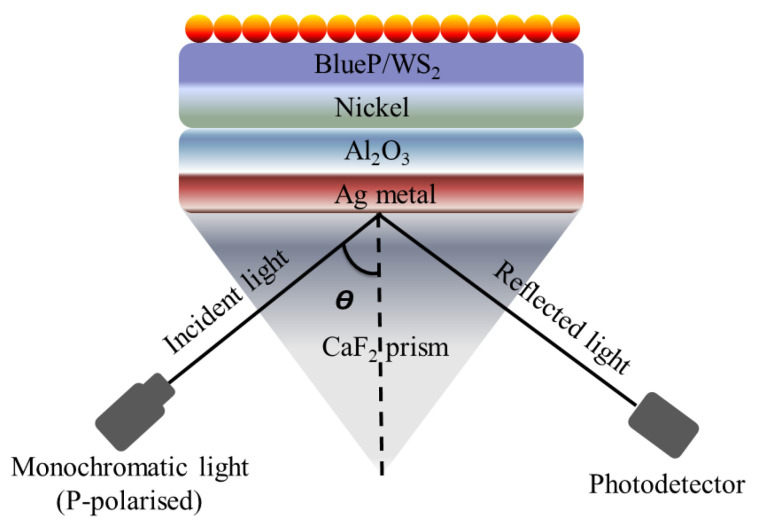
Schematic of the reported surface plasmon resonance (SPR) sensor based on Krestchman configuration [117].

Plasmonics has gained huge interest in the past decade due to its unique characteristics, specifically its low cost, compact size, and ability to confine light in an extremely small area. However, the application of plasmonics is highly restricted by the large propagation loss associated with the plasmonic waveguide. On the other hand, the dielectric waveguides take advantage of low loss, although the mode confinement is relatively weaker [106]. In several reports, the authors utilized the dielectric material composition to improve the sensing response and detection range [107,108]. The sensing performance of the traditional SPR-based biosensors is restricted to 1 pg/mm^2^ surface coverage of target molecules, and consequently, these sensors struggle to detect the interaction of small molecules in low concentrations. To overcome this challenge, Hu et al. reported an SPR-based biosensor using Au nanocluster-embedded $SiO_{2}$ film [109]. With the reported approach, the authors achieve a 10-fold improvement in the resolution and sensing performance. The sensitivity of the fabricated biosensor was observed to be improved by tuning the size and volume fraction of the embedded Au nanoclusters. The obtained sensing response exhibited an ultra-high resolution and detection performance of approximately 0.1 pg/mm^2^ surface coverage of biomolecules. In 2004, Lau et al. reported a planar optical waveguide based on the gold film covered with porous aluminum oxide $\left(porous-Al_{2}O_{3}\right)$ for biochemical sensing [110]. The developed sensor exhibited a high sensitivity to the molecules adsorbed in the bulk of $porous-Al_{2}O_{3}$. Later in 2008, it was ascertained both experimentally and theoretically that the introduction of adsorbate into $porous-Al_{2}O_{3}$ synthesized on aluminum by anodization can play a key role in the sensitivity enhancement of an SPR sensor [111]. In 2015, Jin et al. demonstrated a detailed investigation of such high-index dielectric material and observed an extremely strong surface-plasmonic absorption at the metal-high index dielectric interface [112]. In the reported numerical analysis, the authors compared the performance of multiple high-index dielectric materials, including $TiO_{2},SiO_{2},HfO_{2}\;and\;Al_{2}O_{3}$. The authors observed that the integration of high-index dielectrics with silver exhibited enhanced surface-plasmon absorption because of the quantum-spillover-supported interfacial electron–hole pair production. Later on, several researchers utilized the combination of the metal-high dielectric to enhance the sensitivity [113,114,115]. In 2019, Vahed et al. reported Air/$MoS_{2}$/Nanocomposite/$MoS_{2}$/Graphene to improve the sensing performance [116]. The proposed sensor was based on Otto configuration and achieved a maximum sensitivity of 200^∘^/RIU using six layers of $MoS_{2}$ and a nanocomposite containing gold nanoparticles and $TiO_{2}$ as a dielectric. Most recently, Alotaibi et al. reported a numerical investigation of the enhancement of the sensitivity of an SPR sensor with blue phosphorus/$WS_{2}$-covered $Al_{2}O_{3}$-nickel nanofilms [117]. The $Al_{2}O_{3}$ sheet is sandwiched between silver (Ag) and nickel (Ni) films deposited on the Kretschmann configuration, as shown in Figure 12. The theoretical analysis exhibited an improved sensitivity of $332^{\circ }$ /RIU for the metallic arrangement comprising a thin 17 nm of $Al_{2}O_{3}$ and 4 nm of Ni for refractive indices varying from 1.330 to 1.335. In the reported work, the authors also observed that the sensitivity can be modulated and managed by tuning the thickness of Ni and $Al_{2}O_{3}$.

**Figure 13:**
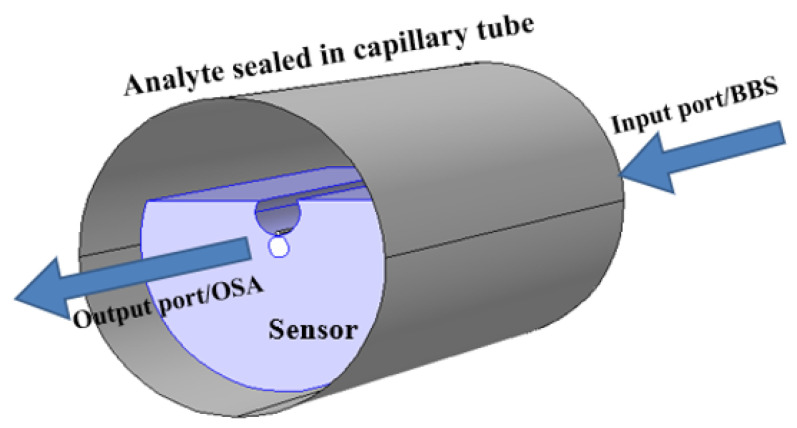
Graphene-coated silver nanowire placed in a microfluidic channel [120].

In some literature, graphene has also been considered as another potential material to improve sensing performance. Graphene is a 2D sheet of single-layer $sp_{2}$-bonded carbon atoms comprised of a structure identical to the honeycomb lattice. Graphene layers have been widely reported in the past decades due to their high surface-to-volume ratio, high electron mobility, and strong absorption. Considering the properties of graphene, Patnaik et al. proposed a D-shaped optical fiber sensor coated with $TiO_{2}$ and graphene [118]. The reported configuration achieved a maximum sensitivity of 5700 nm/RIU. Another work reported by Fu et al. shows an improvement in the sensing performance by considering graphene in multiple silver nanocolumns [119]. The authors achieved a high sensitivity of 8860 nm/RIU compared to the thin film. In 2021, Pathak et al. further enhanced the sensitivity of such a device by utilizing a graphene–silver composite single nanowire placed in a microfluidic channel incorporated within a D-shaped single-mode fiber, as shown in Figure 13 [120]. The authors achieved an improved wavelength and amplitude sensitivity of 13,700 nm/RIU and 1026/RIU, respectively, for small-range analytes varying from 1.330 to 1.350 at a step of 0.005. The obtained result was reported to be two times higher compared to the pure silver nanowire in such a microfluidic channel [121].

Recently, Zhang and Chen utilized a composition of graphene and $Al_{2}O_{3}$ to enhance the sensing response of the photodetector in the near-infrared region [122]. To improve the photocurrent and reduce the dark current of the reported device, the detector of the device was optimized to improve the responsivity of the device. A 2 nm thin $Al_{2}O_{3}$ layer was considered as the passivation layer. The InP layer between the InGaAs and $SiN_{x}$ was retained. The reported configuration comprises a layer of thin silver nanoparticles coated over single-layered graphene. The surface plasmon resonance of Ag NPs leads to the enhancement of the local electric field of the InGaAs interface and enhances the light absorption property of graphene, which can promote carrier generation and transmission in graphene and, therefore, significantly enhance the photocurrent of the device. The device achieved a high responsivity of 265.41 mA/W at 1064 nm and a detection rate of 4.06 ×$10^{11}$ cm H$z^{1/2}$$W^{-1}$. At −1.25 V, the responsivity of the device is improved to 1618.8 mA/W.

In addition to these materials, several researchers reported achieving an improved sensing response using tantalum dioxide ($Ta_{2}O_{5}$) [123,124,125]. Recently, Das et al. reported sensitivity enhancement of a $Ta_{2}O_{5}$-coated photonic crystal fiber (PCF)-based glucose sensor [126]. The reported sensor utilized a new approach to avail external sensing mechanism by introducing the solid core at the fiber periphery. The authors reported an increment in the wavelength sensitivity with the increasing thickness of $Ta_{2}O_{5}$. A maximum sensitivity was reported of 0.81429 nm/g/L for 50 nm thick $Ta_{2}O_{5}$. From the overall discussion, it can be observed that the composition of metal and high-index dielectric material significantly enhances the sensing performance of SPR-based devices. Hence, it can be concluded that the enhancement of surface plasmon resonance (SPR) devices through the incorporation of dielectric materials represents a promising avenue for improving their performance. Dielectric materials play a crucial role in modifying the optical properties of SPR structures, offering unique opportunities to tailor the sensitivity, resolution, and overall efficiency of these devices. By strategically selecting and integrating dielectric materials with specific refractive indices, researchers can effectively control the propagation of surface plasmons, influencing their dispersion characteristics and enhancing the sensitivity of SPR sensors. Additionally, dielectric layers can contribute to minimizing signal loss and increasing the depth of penetration of the evanescent field, leading to improved detection limits and enhanced precision in sensing applications. Furthermore, dielectric materials can be engineered to mitigate issues, such as surface roughness and instability, which are common challenges in SPR devices. Overall, the judicious use of dielectric materials in SPR devices holds great potential for advancing their capabilities, making them more versatile and robust for a wide range of applications in biosensing, environmental monitoring, and other fields.

## 5. Meta-Material-Based Sensing Devices with Improved Sensing Performances

**Figure 14:**
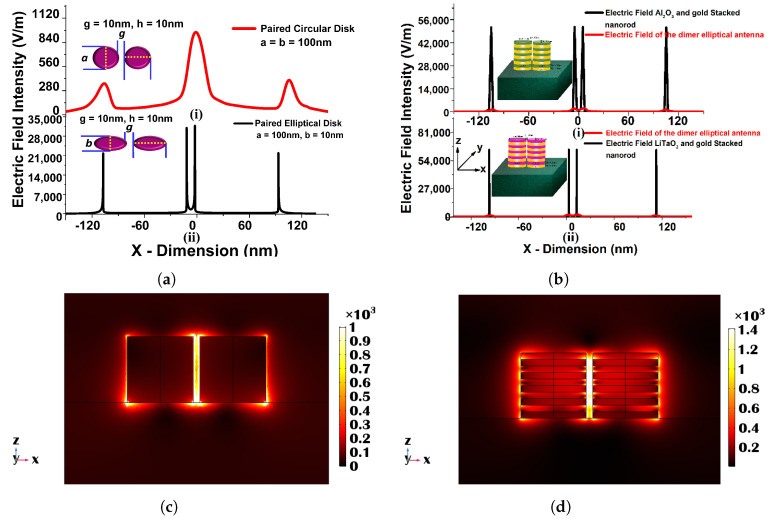
(**a**) Electric field distribution along the x-plane in the (i) circular paired when a=b = 100 nm, *g* = 10 nm, and *h* = 10 and (ii) gold elliptical structure when *a* = 100 nm, *b* = 10, *g* = 10 nm, and *h* = 10. (**b**) Electric field distribution along the x-plane in the single gold and stacked 10 layers (with (i) $LiTaO_{3}$ or (ii) $Al_{2}O_{3}$) elliptical paired structure when *a* = 100 nm, *b* = 10, *g* = 10 nm, and $h_{1}$ = $h_{2}$ = 10 nm. (**c**) $E_{y}$, mode field profile of a single metallic elliptical nanostructure when *a* = 100 nm, *b* = 10 nm, and *h* = 100 nm along the *x*–*z* plane. (**d**) Electric field variation along the *x*–*z* plane for a 10 layered $LiTaO_{3}$ stacked nanostructure when *a* = 100 nm, *b* = 10 nm, and $h_{1}$ = $h_{2}$ = 10 nm [130].

This section reports the increased performance of the metamaterial sensors. In 2009, Kabashin et al. reported the sensitivity of nearly 30,000 nm/RIU for 2D porous gold nanorod arrays on a plasmonic metamaterial stacked with Au and $Al_{2}O_{3}$ with different heights [127]. This was a significant achievement in the field of meta-material sensors. After a few years, Shreekanth et al. developed the grated coupled hyperbolic meta-material sensor and achieved 30,000 nm/RIU sensitivity [128]. Similarly, Yousafi et al. discussed the rectangular patch nanoantenna based on the hybrid plasmonic waveguides [129]. Verma et al. [130] reported a study of a hybrid ($LiTaO_{3}$ and $Al_{2}O_{3}$) stacked metallic nanoplasmonic sensor. The designed and optimized sensor with *a* = 100 nm, *b* = 10 nm, *g* = 10 nm, $h_{1}$= 10 nm, and $h_{2}$ = 10 nm has been evaluated in various surrounding refractive indices from 1.0 to 1.5 to calculate their corresponding sensitivity. The transmission, absorption, reflection spectra, and modal field profiles have also been calculated to observe the sensor performance. The designed hybrid sensor has been compared with a single metallic nanoantenna when *a* = 100 nm, *b* = 10 nm, *g* = 10 nm, and *h* = 100 nm to observe the sensitivity enhancement. From the above-shown results, it can be stated that the sensitivity can be enhanced by nearly 1.5 times by using $Al_{2}O_{3}$-stacked antenna and more than 2 times by using $LiTaO_{3}$, as shown in Figure 14.

With so many benefits that transform their applicability in several industries, metamaterial-based nanoantenna sensors are a revolutionary development in sensing technology. One significant benefit is their capacity to regulate and modify electromagnetic waves at the nanoscale, which makes it possible to detect minute changes in the environment with previously unheard-of sensitivity and specificity. The design flexibility of metamaterials makes it possible to customize the antenna characteristics to certain frequencies, improving the selectivity of the sensor and making it possible to detect a broad variety of target molecules or signals. These nano-antennas also show impressive miniaturization, which makes it possible to incorporate them into small devices for wearable and portable applications. Their effective energy-harvesting qualities add to longer-lasting sensors and lower power usage. By providing subwavelength resolution, metamaterial-based sensors also excel at overcoming conventional constraints like the diffraction limit, hence improving imaging capabilities. Furthermore, their adjustable qualities enable in-the-moment modifications to conform to changing surroundings, guaranteeing adaptability in sensing uses. For the most part, metamaterial-based nano-antenna sensors are promising as a technology for the future generation of sophisticated sensing devices because of their benefits in terms of higher sensitivity, selectivity, miniaturization, energy efficiency, and flexibility. Hence, meta-material-based sensing devices represent a groundbreaking advancement in the field of sensor technology, offering unparalleled improvements in sensing performances. Meta-materials, engineered to exhibit unique electromagnetic properties not found in nature, enable these devices to manipulate and control electromagnetic waves with precision. This capability opens the door to a wide range of applications, from enhanced imaging to improved communication systems. The tailored design of meta-materials allows for the creation of sensors with increased sensitivity, selectivity, and resolution. By carefully engineering the composition and structure of meta-materials, researchers can tune the devices to specific frequencies, resulting in superior detection capabilities. Additionally, the ability to control the propagation of electromagnetic waves enables the development of compact and efficient sensing devices, making them suitable for diverse environments and applications. As we delve deeper into the realm of meta-material-based sensing, the potential for groundbreaking innovations in healthcare, environmental monitoring, and security applications becomes increasingly evident. The ongoing research and development in this field hold promise for a future where sensing devices play a pivotal role in addressing complex challenges across various domains.

## 6. Surface Plasmons Resonance for Biosensing Applications

The demand for contemporary biomedical care and the importance of physical tests are both rising along with the improvement in living standards. Early intervention and treatment of diseases via physical examination are expected to yield better results. The primary task of achieving this goal is the detection of biomarkers related to various diseases. Several optical plasmonic-based biosensors have been demonstrated in past decades to achieve the goal of real-time detection of biomolecules, proteins, and viruses [131,132]. These plasmonic sensors can be categorized based on the location of the metal deposition and are hence known as external and internal sensing [133]. In the case of external sensing, the plasmonic layer and analytes remain outside the fiber, e.g., D-shaped, etched, U-bent fibers, etc. [134], whereas in internal sensing, all these arrangements remain inside the substrate, such as metal-deposited photonic crystal fiber.

### 6.1. Planar Waveguide SPR Biosensors

Biosensors utilizing SPR features in planar structures are widely recognized for their ease of fabrication and their capability to detect multiple viruses simultaneously [135]. Among several viruses, the most commonly detected viruses by the planar-structure biosensors are influenza A and B, parainfluenza viruses 1–3 (PIV1, 2, 3), avian influenza virus, Ebola virus, Dengue virus, adenovirus, H1N1, hepatitis B, respiratory syncytial virus (RSV), and one of the recently recognized of coronavirus, the severe acute respiratory syndrome (SARS) virus [136,137]. All the reported planar biosensors utilized a similar configuration of having a plasmonic layer topped with a virus binding layer for target-specific detection. To measure the SPR of the planar biosensor, the momentum of the incident photon and the conduction band electrons are measured. If both signals coincide and the analyte media has a high refractive index, light is coupled, forming attenuated total reflection (ATR). This could be interpreted by using the dispersion relation given by Raether in 1988 [138].

**Figure 15:**
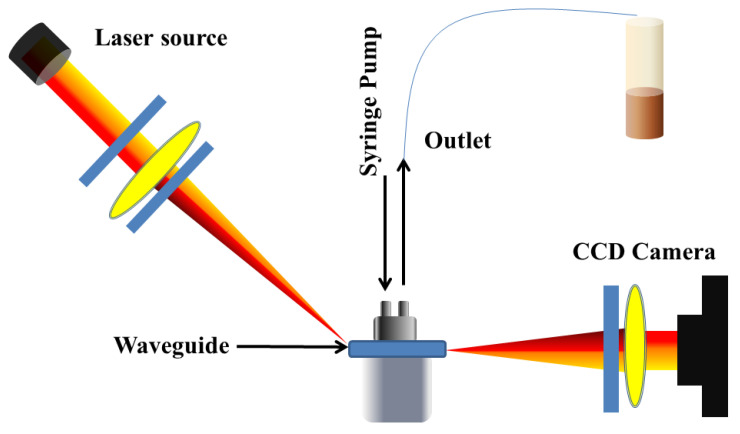
Designs of planar waveguide. Reprinted/adopted from [139].

In 2018, Jawdah et al. developed a simple and highly sensitive optical biosensor for the detection of mycotoxins [139]. This reported sensor was built on a planar waveguide as shown in Figure 15. The reported design operates on the polarization interferometry principle, i.e., it monitors the phase shift between p- and s-components of polarized light that appears during the binding of analyte molecules. This planar polarization interferometer has a sensitivity of 5200 rad/RIU. A series of biosensing experiments have been conducted for detecting ochratoxin A in direct immunoassay with specific antibodies. This biosensor was capable of detecting 0.01 ng/mL of ochratoxin A. The sensing principle of the device was similar to that of a Mach–Zehnder interferometer [108], while this design is much simpler and does not require splitting the waveguide into two arms. The refractive index sensitivity of the polarization interferometer sensor was in the range of 5200 radians per refractive index unit (RIU). Multiple tests have been reported on the device to detect ochratoxin A at various concentrations in direct immunoassay with specific antibodies immobilized in the sensing window. The lowest concentration of ochratoxin A of 0.01 ng/mL results in a phase shift of nearly one period. The results obtained prove the high sensitivity of the sensors, which were reported to be capable of detecting even lower concentrations of mycotoxins at the part-per-trillion (ppt) level.

In 2019, Konopsky and Alieva reported a biosensing technique with a two-dimensional spatial resolution based on a planar optical waveguide [140]. The thickness of the waveguide was selected in such a way that the wavelength of the waveguide mode was cantered between the maxima of the blue and green pixels of the color camera. Waveguide excitation was achieved using a prism-based Kretschmann-like setup, positioned between crossed polarizers to acquire the waveguide resonance peak. The spectral shift in the resonance peak was determined by analyzing the normalized differences in intensities between the blue and green pixels on the color camera. The spatial distribution of resonance wavelength shifts across the camera corresponded to changes in the thickness of the adsorbate over the sample. This biosensor’s sensitivity, dynamic range, and practical implementation were compared with those of an imaging biosensor based on photonic crystal surface mode. The reported biosensor showed 1.5–2.7 times better sensitivities and lower baseline noises compared to the similar photonic crystal surface mode biosensor. Nonetheless, the waveguide biosensor exhibited a dynamic range that was 1.5–2.7 times less effective, and its practical implementation was complicated by the high refractive index of the utilized prism.

**Figure 16:**
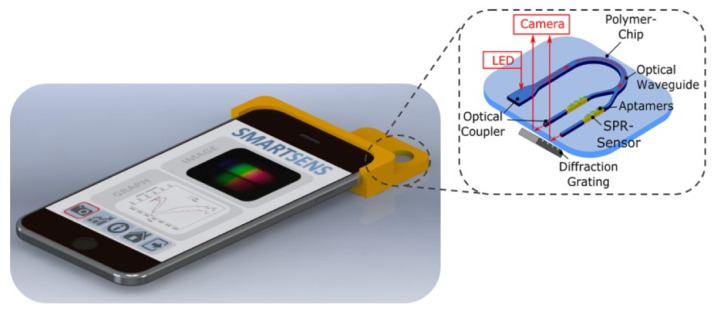
Schematic of the all-optical planar polymer-based biochip sensor platform. Reprinted/adopted from [141].

In 2020, Walter et al. reported an all-optical SPR sensor designed for smartphones based on planar-optical waveguide configuration integrated into a polymer chip, as shown in Figure 16 [141]. The sensor system’s applicability for biosensing purposes was demonstrated by detecting 25-hydroxyvitamin D (25OHD) in human serum samples using an AuNP-enhanced aptamer-based assay. The fabricated assay exhibited a good sensitivity of 0.752 pixel/nM for 25OHD concentrations varying from 0 to 100 nM. The sensor’s waveguide structure enabled miniaturization and parallelization, showcasing the potential for the simultaneous detection of various analytes, including biomarkers. The entire optical arrangement could be integrated into a single polymer chip, allowing for large-scale and cost-efficient sensor fabrication. The reported sensor was highly attractive for wider use in lab-on-chip applications due to the broad utilization and accessibility of smartphone electronics.

**Figure 17:**
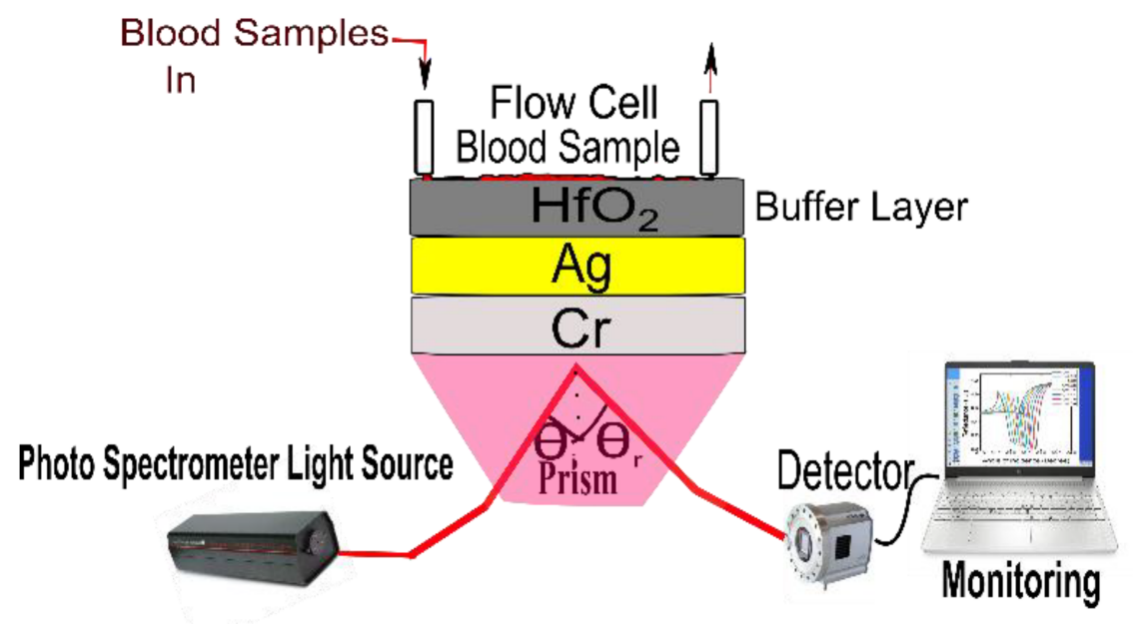
Schematic of the proposed prism-based surface plasmon resonance (SPR) biosensor for identifying human blood groups. Reprinted/adopted from [142].

In 2023, Pandey et al. reported the potential of chromium (Cr), silver (Ag), and hafnium oxide ($HfO_{2}$) as an SPR biosensor for precise blood-group identification [142]. In the reported work, the author utilized a buffer layer on top of SPR active metal to avoid oxidation and contamination of blood samples, as shown in Figure 17. This study developed a theoretical model using experimental data, taking into account the refractive index of blood samples. The research identifies the BK7 prism as the ideal substrate material for precise blood type identification analysis when utilizing a combination of Ag and Cr as the active SPR metals. The sensor’s performance is thoroughly examined, considering an angular shift, curve width, and other design aspects crucial for accurate blood group identification. Additionally, the study explores the SPR dip slope, detection accuracy, and the figure of merit (FOM) to enhance the potential for future biosensor applications.

Till now, several studies have been made to improve the sensitivity and optimize the LOD of planar biosensors [143,144,145]. These planar waveguide-based biosensors are common to being highly sensitive, compact in size, label-free, and have multiplexed processing [146,147]. They also permit a simple, fixed-wavelength read-out, which makes them suitable low-cost diagnostic monitoring devices.

### 6.2. Cylindrical Waveguide SPR Biosensors

A plasmonic optical fiber-based sensor generally relies on the phase-matching conditions that take place between the surface plasmon polariton (SPP) and the guided mode, commonly known as the SPR condition [148,149]. This phenomenon occurs when the wave vector of the optical propagating wave within the fiber aligns with the propagation constant of SPP. This alignment is highly sensitive toward changes in the surrounding medium. Furthermore, when photons excite the metal–dielectric interface, they generate surface plasmons. During the resonance condition, the surface plasmon waves (SPW) at the metal interface predominantly extract energy from the optical wave photons at a specific wavelength, resulting in a maximum loss of the optical wave as it propagates. Consequently, the variation in the surrounding environment leads to changes in the resonance wavelength, facilitating sensitive sensing. The sensitivity of this sensor is quantified as the ratio between the change in refractive index and the corresponding shift in resonance wavelength. In recent years, several optical fiber-based SPR biosensors with outstanding performance have been documented in the scientific literature [150,151,152].

#### 6.2.1. Standard Fiber Biosensor

**Figure 18:**
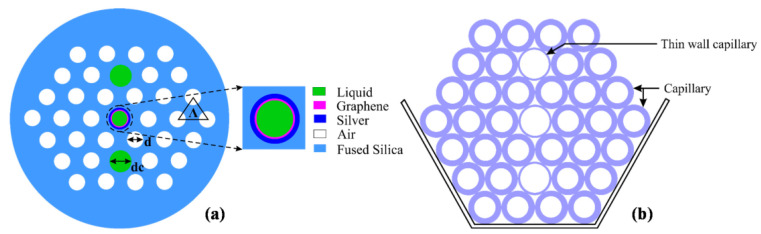
(**a**) Cross-section of the proposed sensor. (**b**) Cross-section of the stacked preform. Reprinted/adopted from [153].

In 2015, Rifat et al. designed a photonic crystal fiber-based SPR sensor with a selective filling of analyte channels and graphene–silver deposited core, as shown in Figure 18 [153]. In the reported work, the author considered silver as the plasmonic material for the accurate detection of the analytes, which was later coated with a thin graphene layer to prevent oxidation. The liquid-filled cores were considered near the metallic channel for easy excitation of free electrons to produce SPWs. The numerical investigations of the sensing properties and sensing performance were performed using the finite element method (FEM). The reported sensor shows a maximum amplitude sensitivity of 418/RIU with a resolution as high as $2.4\times 10^{-5}$/RIU. Using the wavelength interrogation method, a maximum refractive index sensitivity of 3000 nm/RIU in the sensing range of 1.46–1.49 is achieved. The proposed sensor was reported to be suitable for detecting various high refractive index chemicals, and biochemical and organic chemical analytes.

**Figure 19:**
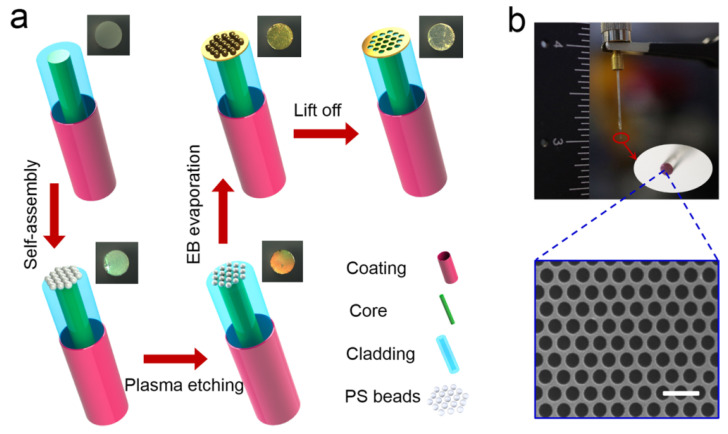
(**a**) Schematic of the bottom-up fabrication procedure for nanohole array-based optical fiber probe. (**b**) A photograph of the optical fiber sensing probe. Reprinted/adopted from [155].

In 2018, Zhan et al. reported a numerical analysis of a novel SPR microfiber sensor covered with gold nanowires to enhance the sensitivity [154]. In the reported work, the author compared the performance of the gold nanowire-covered device thin film. The gold nanowires exhibited significant enhancement in the performance of the sensor due to the effect of localized SPR. The influence of the diameters of gold nanowires and microfiber on the sensing properties is investigated and optimized by using the FEM. The sensor exhibited a maximum sensitivity of 5200 nm/RIU for fiber diameters of 3 μm and nanowire of diameter 120 nm, along with the FOM of 150.38/RIU. Both the sensitivity and the FOM were observed to be improved when the refractive indices increased from 1.33 to 1.40. For refractive index 1.40, the sensor showed an extremely high sensitivity of 12,314 nm/RIU. In 2019, Liang et al. fabricated a plasmonic nanohole patterned on the tip of multimode optical fiber by self-assembly nanosphere lithography technique, as shown in Figure 19 [155]. The technique used for nanofabrication and nanotechnology for creating periodic nanostructures. The high numerical aperture and large facet area of multimode fiber not only simplify the fabrication process but also improve the coupling efficiency. The plasmonic fiber nanoprobes feature a clear reflectance inclination and a potent near-field electromagnetic amplification as a result of the resonance coupling of the plasmonic mode. The sensor performance of the plasma fiber optic nanoprobe was investigated on various refractive indices such as 1.3333, 1.3451, 1.3564, 1.3675, and 1.3785 prepared by dissolving sodium chloride (NaCl). The fabricated sensor exhibited a good refractive index sensitivity of 432 ±21 nm/RIU.

**Figure 20:**
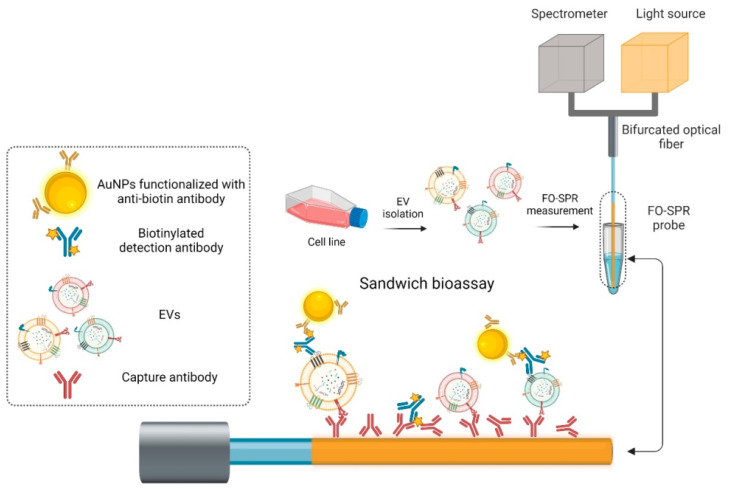
Schematic of the different steps from the FO-SPR EV detection sandwich bioassay. Reprinted/adopted from [156].

Recently, in 2023, Yildizhan et al. reported the strategy of fabrication and detection of breast cancer-specific extracellular vesicles using fiber optic SPR biosensors [156]. The study focuses on the potential of using extracellular vesicles (EVs) as cancer biomarkers. Many existing EV detection technologies are unsuitable for clinical use due to their complexity, lack of sensitivity, specificity, or standardization. To address this, the researchers have created a highly sensitive breast cancer-specific EV detection assay directly in blood plasma. In the reported work, the authors utilize a fiber-optic (FO) SPR biosensor, which was calibrated with recombinant EVs. The fabrication and functionalization of the FO-SPR sensor are shown in Figure 20. The assay was established by functionalizing sensing configuration with anti-HER2 antibodies to detect SK-BR-3 EVs. A calibration curve was constructed, resulting in a limit of detection (LOD) of 2.1 × $10^{7}$ particles/mL in buffer and 7 × $10^{8}$ particles/mL in blood plasma. The assay was also tested for the detection of MCF7 EVs in blood plasma using an anti-EpCAM/anti-mix combination, achieving an LOD of 1.1 × $10^{8}$ particles/mL. Importantly, the assay exhibited specificity by not yielding a signal when testing plasma samples from 10 healthy individuals without breast cancer. This highly sensitive and specific sandwich bioassay, combined with the standardized FO-SPR biosensor, holds significant promise for the future of EV analysis.

#### 6.2.2. Side-Polished Optical Fiber SPR Biosensors

Polishing the cladding section of a standard optical fiber to form a D shape appears as an alternative approach for fabricating a miniaturized plasmonic fiber biosensor. Such configuration includes precise polishing of cladding to excite the evanescent field [157], which was later coated with plasmonic materials. The interaction between the fiber and plasmonic modes can form an absorption peak (or transmission dip) in transmission spectra based on the interaction of measurement with the plasmonic materials.

**Figure 21:**
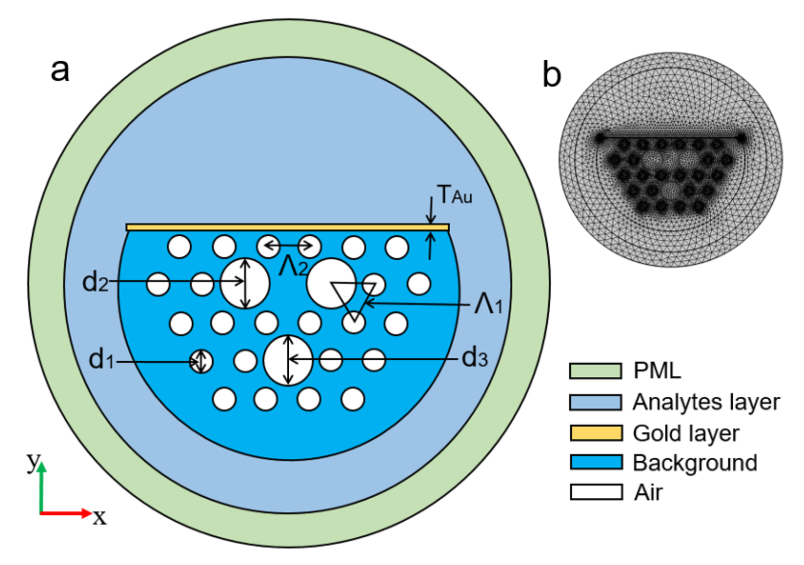
(**a**) Schematic of a cross-section of the sensor; (**b**) meshing of the proposed design. Reprinted/adopted from [159].

In 2019, Dong et al. experimentally demonstrated the potential of a side-polished few-mode fiber as an SPR biosensor using a thin layer of gold on a polished surface [158] following numerical optimization. Using optimization techniques, the researchers achieved a maximum sensitivity of 4903 nm/RIU for the refractive index varying from 1.333–1.404 and the FOM of 46.1/RIU. The sensors also exhibited an excellent bovine serum albumin (BSA) refractive index sensitivity of 6328 nm/RIU and an averaged BSA concentration sensitivity of 1.17 nm/(mg/mL). The use of the few-mode fiber led to narrower SPR spectra and superior FOM. In 2020, Chen et al. numerically reported a D-shaped photonic crystal fiber (PCF)-based plasmonic sensor, as shown in Figure 21a [159]. Figure 21b shows the meshing of the proposed design used for the simulation. Numerical results presented showed that the designed fiber is especially suitable for sensing. The performance of the sensor was investigated in the mid-infrared range varying from 2.9 to 3.6 μm and exhibited a maximal wavelength sensitivity of 11,500 nm/RIU with a maximum refractive index resolution of $8.7\times 10^{-6}$ RIU for the analyte varying from 1.36 to 1.37. Along with the sensitivity, the author also investigated the fabrication tolerance of the device, which shows good tolerance when the structural parameter was varied by ±3% from its optimized value.

**Figure 22:**
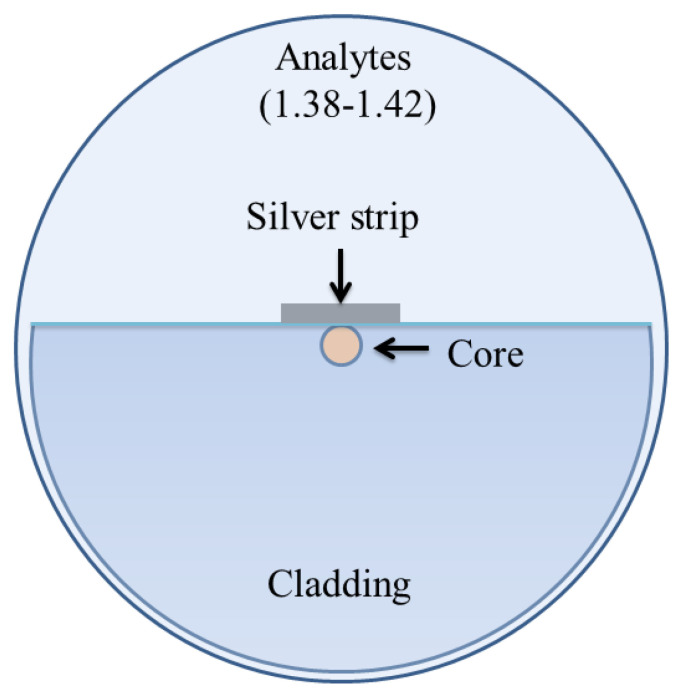
Schematic diagram of the nanoscale silver strip sensor. Reprinted/adopted from [160].

In 2020, Pathak and Singh reported a D-shaped optical fiber chemical sensor coated with a nanoscale silver strip, as shown in Figure 22 [160]. The sensing property of the design was investigated using FEM based on COMSOL multiphysics. An average wavelength sensitivity of 2100 nm/RIU was reported for a wide range of refractive index varying between 1.34 and 1.42 as an enhanced wavelength and amplitude sensitivity of 3240 nm/RIU and 192/RIU was observed for a higher refractive index varying between 1.38 and 1.42. The results exhibited that the proposed sensor performs in both high and low refractive indices, while a significant enhancement in sensitivity was observed for higher refractive index solutions; hence, it can be potentially used for chemical and biological sensing.

**Figure 23:**
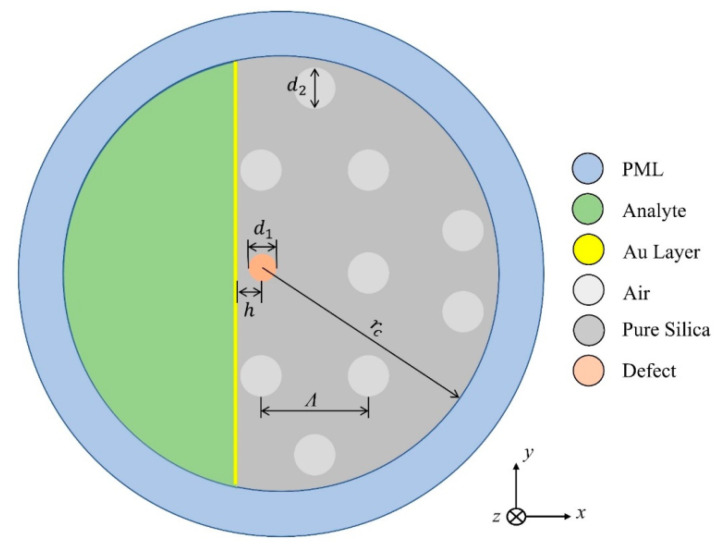
Schematic of a cross-section of the sensor design. Reprinted/adopted from [161].

In 2022, Cunha and Silva reported another interesting study of the germanium dioxide ($GeO_{2}$)-doping defect on the sensitivity of a D-shaped PCF sensor [161]. The investigation was carried out on the doping of a silica ($SiO_{2}$) core with various concentrations of $GeO_{2}$. In this work, the author considered gold-coated D-shaped PCF, which was polished up to the core of the fiber, which was doped with various concentrations of $GeO_{2}$, as shown in Figure 23. The performance of the device was investigated for a wide range of analytes, varying from 1.32 to 1.45, using the FEM. The obtained result exhibited that the highest sensitivity of 12,133.47 nm by RIU was obtained without any doping, and it was observed that the sensitivity of the device decreases with increasing concentrations of $GeO_{2}$. The lowest average sensitivity of 9229.90 nm/RIU was reported for a structure with a 19.3% concentration of $GeO_{2}$ (19.3%).

### 6.3. Novel-Designed Optical Fiber SPR Biosensors

Novel designs of optical fiber sensors represent a cutting-edge frontier in the realm of sensing technology. These novel-designed sensors employ innovative configurations and techniques to significantly enhance their sensitivity, precision, and versatility. By miniaturizing and adapting their designs, optical fiber sensors can be seamlessly integrated into a wide array of applications, spanning from healthcare and environmental monitoring to industrial automation. Their capacity to detect diverse parameters, such as temperature, pressure, chemical compounds, and specific biomolecules, makes them invaluable tools for addressing complex challenges across various industries.

**Figure 24:**
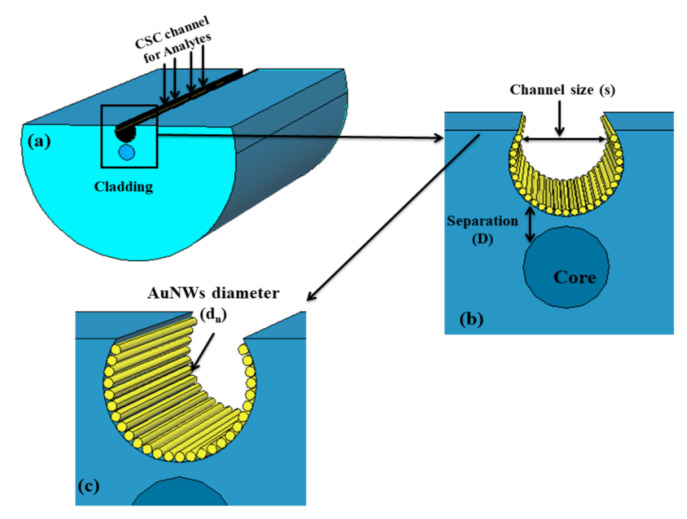
Schematic diagram of the designed concave-shaped localized surface plasmon resonance (LSPR)-based refractive index sensor (**a**) cross section of designed sensor, (**b**,**c**) are the zoomed in and further magnified diagram of gold nanowires (AuNWs) covered concave shaped channel (CSC), respectively. Reprinted/adopted from [162].

Pathak and his group reported a series of numerical investigations on the external sensing approach using a novel design of optical fiber facilitating the SPR phenomenon. The reported biosensors were investigated using the FEM to achieve extremely high sensitivity, reduced cost, and provide a user-friendly interface. In 2019, Pathak et al. performed a comparative analysis on thin gold film and gold nanowires placed in a concave-shaped optical fiber, as shown in Figure 24 [162]. The influence of structural parameters, such as the distance of the concave-shaped channel from the core, the diameter of the gold nanowires (AuNWs), and the size of the sensor, was investigated and optimized to attain the maximum response. It was observed from the analysis that the AuNWs exhibited significant improvement in sensitivity and performance compared to the Au thin film. The AuNWs-filled sensor exhibited an enhanced sensitivity of 4471 nm/RIU for a wide range of analytes varying from 1.33 to 1.38. However, for conventional Au film, a sensitivity of 808.57 nm/RIU was obtained for the same range of analytes.

In 2020, Esfahani [157] reported a D-shaped SPR photonic crystal sensor coated with titanium nitride (TiN) due to its excellent melting point, chemical stability, conductivity, and compatibility with complementary metal-oxide semiconductor (CMOS) technology. The sensor structure comprises a D-shaped PCF structure with the analytes filled in the central core, surrounded by several air holes within the silica background. The sensor exhibited a maximum spectral sensitivity of 16.275 nm/RIU with an amplitude sensitivity of 206.25/RIU and a maximum FOM of 147.9/RIU. The reported device can effectively detect a refractive index ranging from 1.44 to 1.52. The authors have shown that TiN performs comparably to gold-based PCF sensors and is suitable for applications in chemical and biological sensing. In the same year, Han et al. reported an H-shaped SPR PCF sensor for a large detection range varying from 1.33 to 1.49 [163].

**Figure 25:**
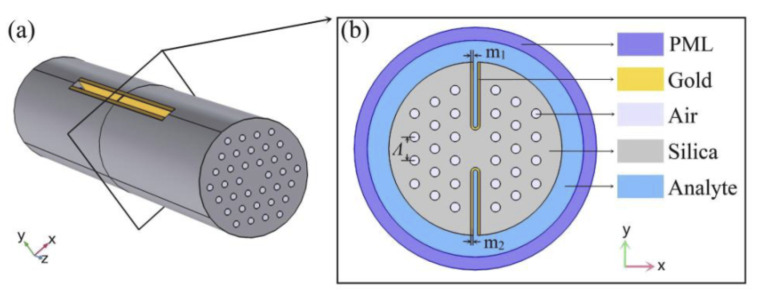
(**a**) Schematic diagram of the proposed H-shaped photonic crystal fiber (PCF)-surface plasmon resonance (SPR) sensor; (**b**) cross-section of the SPR sensor. Reprinted/adopted from [163].

Figure 25a shows the schematic of the proposed fiber whereas (b) shows the cross-section of the design. In the reported work, the authors considered two deep U-shaped grooves opposite to each other, hence forming an H-shaped PCF. The grooves of the H-shaped PCF were working as the sensing channels, which were coated with a 40 nm-thin gold film. The sensing performance of the proposed sensor was investigated using FEM. The numerical results exhibited the highest sensitivity of 25.900 nm/RIU for analytes ranging from 1.47 to 1.48, whereas they show an average sensitivity for refractive indices of 1.33 to 1.49. Additionally, the author also reported excellent tolerance when the structural parameter varied by ±10% from its optimized value during fabrication.

Later in 2021, Pathak et al. achieved three times higher sensitivity from a similar design but using a single silver nanowire functionalized with a graphene layer [120]. In the reported work, the author utilized graphene on top of silver nanowires for three reasons: (i) to prevent oxidation, (ii) to improve sensitivity, and (iii) to make it compatible with biomolecule detection. The sensing performance and the coupling properties of the designed sensor were numerically analyzed in the detection range, which varied from $n_{a}$ = 1.330–1.350 at a step of 0.005. The sensor exhibited a maximum wavelength and amplitude sensitivity of 13,700 nm/RIU and 1026/RIU, respectively.

**Figure 26:**
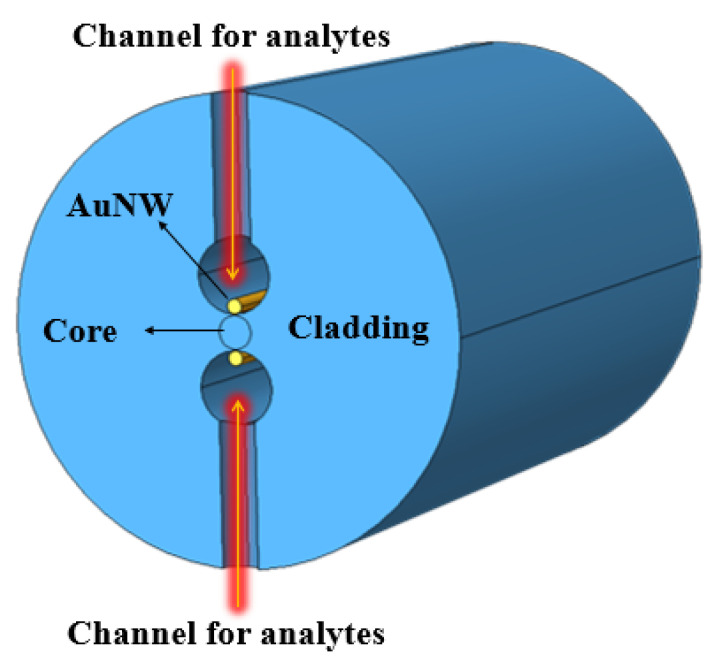
Dual drilled channel filled with AuNW.

In the same year, Tulika and Singh reported a V-groove fiber plasmonic sensor with facile resonance tenability. In the reported work, the authors considered a V-groove channel embedded in the cladding and considered a gold nanowire of radius 0.3 at the base of the channel. The V-groove channel in the reported design acts as a microfluidic channel and the core itself when filled with analytes of high refractive index. The performance of the device was analyzed using FEM, and the obtained results exhibited the highest wavelength sensitivity of 20.400 nm/RIU along with an amplitude sensitivity of 700/RIU for the analyte refractive index ranging from 1.455 to 1.5. In 2022, Pathak et al. designed a dual channel in SMF considering a single AuNW in each channel and achieved a higher sensitivity [164] than any of the previously reported sensors [121,132,133,160,165]. The schematic diagram of the designed sensor is shown in Figure 26. The sensor comprised a dual-drilled channel with AuNW in each channel to excite the plasmon modes. The sensing performance, coupling characteristics, and fabrication tolerance of the proposed design were numerically investigated using an FEM technique. The designed sensor exhibited an average wavelength sensitivity of 3150 nm/RIU for refractive index = 1.310, while an extremely high sensitivity was obtained of 90,500 nm/RIU for a refractive index ranging from 1.370 to 1.400. The achieved sensitivity of the reported design makes it a potential candidate in chemical and biological sensing for various biofluids, including glucose monitoring, immunoglobulin G and M monitoring, and blood component detection, such as red blood cells, hemoglobin, etc.

## 7. Future of Plasmonic Sensors

In this section, we will discuss the future perspective of plasmonic sensors. In the earlier section of this paper, we discussed most of the key available plasmonic sensors and the different materials that can enhance their performance. Finally, the applications and expected future breakthroughs of plasmonics are summarized in Table 2.

The above table provides a brief overview of the future applications in plasmonics. The new discovery of plasmonics will have a significant impact on the design of future plasmon-based devices, as it paves the way to control the electrical excitation of plasmonic nanostructures down to, and even below, the level of an individual molecule, and can allow the direct integration of plasmonic nanostructures into conventional electronic circuits. However, there are numerous breakthroughs of the plasmonics, which are discussed below in detail in Table 3.

Plasmonics is also a promising emerging technology that attempts to put together the best of two worlds—optics and electronics—to achieve faster computation and communication by making optical devices significantly smaller. In recent research, a team of European scientists has solved a long-standing problem in this field by sending signals over a long distance in a breakthrough that brings this technology much closer to mass production.

## 8. Conclusions

In this review article, we have discussed the principle of surface plasmon resonance, which received a lot of attention when it comes to nano-structured devices. A fair comparison has been made between the traditional antenna and the nano-structured antenna. The occurrence of surface plasmonic devices has been discussed in terms of light–matter interactions. The Drude model has also been discussed to understand the nature of free electrons present in metallic structures in terms of the dielectric constant. In this review paper, we have also shown the performance of the metallic structured device in terms of scattering, absorption, and extinction cross-sections. The effect of the structural change has also been discussed and plotted in this paper, just to observe the performance of the nano-structured device. The effect of the paired disk and elliptical antenna has been plotted to calculate the sensitivity and electric field confinement. This paper also discusses the enhancement in sensitivity when the dielectric material is used in the antenna device. Finally, this review wrapped up with several bio-sensing applications, future scopes, and breakthroughs in plasmonics.

## Figures and Tables

**Table 1:** List of the various nanostructural shapes and corresponding sensitivity.

S. No.	Designed Antenna (nm)	Full-Width Half-Maximum (nm)	Sensitivity (nm/RIU)	Ref.
1.	Cubic antenna	125.0985	—	[44]
2.	Circular disk antenna	147.7624	—	[45]
3.	Bow-Tie array antenna	280.4914	—	[46]
4.	Circular disk antenna	109–113	—	[47]
6.	Nanoshell antenna	—	60	[48]
7.	Bipyramids, nanorods, and cubic antenna	—	195–288	[49,50]
8.	Silver nanoantenna	—	200	[51]
9.	Cubic antenna	—	167–327	[52]
10.	Nanodisk antenna	—	200–350	[53]
11.	Nanotube antenna	—	250	[54]
12.	Elliptical Antenna	95–100	510–530	[43,55]

**Table 2:** Future of the plasmonics.

S. No.	Future Perspectives of Plasmonics
1.	Optical nanodevices, optical nanocircuits
2.	Spectroscopic nanoimaging (mainly Raman scattering) characterization and inspection of semiconductors, analysis and evaluation of nanomaterials, bio-imaging, molecular imaging
3.	Highly-sensitive highly-efficient optoelectronic devices (solar cells, light emitting diodes, lasers)
4.	Highly-functional optical materials (optical catalysts)
5.	Nanophotolithography, nanofabrication
6.	Analytical sensors and medical diagnosis and therapy (surface-plasmon sensors, DNA chips, biochips, cancer therapy)
7.	Holography

**Table 3:** Breakthroughs in plasmonic devices.

S. No.	Breakthroughs in Plasmonic Devices
1.	Applications in deep-UV range
2.	Achieving resolutions of 1 and 0.1 nm
3.	Development of nonlinear plasmonics
4.	Development of AI-based plasmonic devices [166,167]
5.	Development of hybrid plasmonic devices for various applications [168]
